# Medical Department, Lind University

**Published:** 1860-12

**Authors:** 


MEDICAL DEPARTMENT, LIND UNIVERSITY.—We understand from the best sources of information accessible to us, that the number of undergraduate *Medical* students in attendance upon the present session of this institution is not far from fifteen.

